# A Retrospective Longitudinal Cohort Study of Antihypertensive Drug Use and New-Onset Diabetes in Taiwanese Patients

**DOI:** 10.1155/2013/287696

**Published:** 2012-12-23

**Authors:** Ching-Ya Huang, Tsochiang Ma, Liyun Tien, Yow-Wen Hsieh, Shwu-Yi Lee, Hung-Yi Chen, Gwo-Ping Jong

**Affiliations:** ^1^Department of Pharmacy, China Medical University Hospital, Beikang Campus, Taichung 40402, Taiwan; ^2^Institute of Pharmacy, China Medical University, Taichung 40402, Taiwan; ^3^Department of Health Services Administration, China Medical University, Taichung 40402, Taiwan; ^4^Central Region Branch, Bureau of National Health Insurance, Taichung 40709, Taiwan; ^5^Yuan Rung Hospital, Changhua 51043, Taiwan; ^6^Division of Internal Cardiology, Armed Forces Taichung General Hospital, and Basic Science, Central Taiwan University of Science and Technology, Taichung 41168, Taiwan

## Abstract

Antihypertensive drugs have been linked to new-onset diabetes (NOD); however, data on the effect of these drugs on the development of NOD in hypertensive patients has not been well determined in a clinical setting. The aim was to investigate the association between antihypertensive drugs and NOD in Taiwan. We conducted a retrospective study of hypertensive Taiwanese patients receiving antihypertensive drugs treatment between January 2006 and December 2011. Clinical information and laboratory parameters were collected by reviewing the medical records. We estimated the odds ratios (ORs) of NOD associated with antihypertensive drug use; nondiabetic subjects served as the reference group. A total of 120 NOD cases were identified in 1001 hypertensive patients during the study period. The risk of NOD after adjusting sex, age, baseline characteristics, and lipid profiles was higher among users of thiazide diuretics (OR, 1.65; 95% confidence interval (CI), 1.12–2.45) and nondihydropyridine (non-DHP) calcium channel blockers (CCBs) (OR, 1.96; 95% CI, 1.01–3.75) than among nonusers. Other antihypertensive drug classes were not associated with risk of NOD. Our results show that patients with hypertension who take thiazide diuretics and non-DHP CCBs are at higher risk of developing NOD than those who take other classes of antihypertensive drugs in Taiwan.

## 1. Introduction

Diabetes mellitus is a major global public health problem, and it is associated with an estimated annual cost of US$174 billion in the USA alone [1, 2]. Concerns regarding new-onset diabetes (NOD) have been raised because of the economic burden it poses in various countries [3]. Recently, some multiple prospective trials of treatments for hypertension initiated a debate about the clinical impact of NOD in hypertensive patients [4–8]. It seems obvious that cardiovascular risk is increased when diabetes and hypertension coexist than when the two conditions stand alone; however, data from these studies are limited due to clinical trials [5, 6] or head-to-head comparisons of drugs [9, 10]. In particular, it is not completely clear whether certain antihypertensive drug classes are associated with higher risk of NOD. Our previous article [11] provided an estimate of the effects of antihypertensive drugs on the development of NOD from the data of the Bureau of National Health Insurance in Taiwan from January 2002 to December 2007. This data suggests that while hypertensive patients who took angiotensin-converting enzyme (ACE) inhibitors, angiotensin receptor blockers (ARBs), or alpha-blockers were at a lower risk of NOD, diuretics, beta-blockers, and calcium-blockers were associated with a significant increased risk of NOD. However, many reports have provided conflicting results about the effects of antihypertensive drugs on NOD under various conditions [12, 13]. Therefore, we conducted another retrospective cohort study to explore the relationship between antihypertensive drugs and NOD in a clinical setting.

The aim of this paper is to determine the effect of antihypertensive drugs [thiazide diuretics, beta-blockers, dihydropyridine (DHP) calcium channel blockers (CCBs), nondihydropyridine (non-DHP) CCBs, alpha-blockers, vasodilators, ACE inhibitors, and ARBs] on NOD in a clinical setting.

## 2. Materials and Methods

### 2.1. Subjects

Our data were taken from medical records provided to the China Medical University Hospital from January 2006 to December 2011. By medical record (electronic chart) review method, selected patients were further clarified to see if they fulfilled the inclusion and exclusion criteria. Electronic chart review contains information regarding patient identification numbers, sex, age, diagnostic codes, current smoking, familial history of diabetes mellitus (DM), body mass index (BMI), blood pressure, total cholesterol, triglyceride, low-density lipoprotein (LDL) cholesterol, high-density lipoprotein (HDL) cholesterol, fasting blood glucose, serum creatinine, and drugs prescription's information. The LDL cholesterol level was obtained by calculation from Friedewald equation, LDL cholesterol = total cholesterol – HDL cholesterol – (triglyceride/5). Due to the influence of Taiwan BNHI policy, the majority of LDL levels were obtained by calculation instead of direct measurement. The prescription table contains the quantity and expenditure for all drugs, operations, and treatments. Patients were included in the study if they had hypertension only without diabetes at baseline (January 1, 2006). We summarized the medical records of each patient into one record.

### 2.2. Study Procedure

We used the International Classification of Diseases, Ninth Revision (ICD-9) Clinical Modification code to define hypertension (ICD-9 codes 401–405) and diabetes (ICD-9 codes 250). Any patient with a diabetes diagnosis or prescription for antidiabetic drugs during 2 years prior to their antihypertensive prescription on January 1, 2004 was excluded. The primary endpoint was NOD, which was the first time that a diabetes code or antidiabetic prescription appeared in the medical records. We identified all prescriptions for antihypertensive drugs administered to patients with and without NOD within a 6-year period before the date NOD was diagnosed. Patients who had used only one type of antihypertensive drug in the 180 days before the date NOD was diagnosed were categorized according to the antihypertensive drug class that they took: thiazide diuretics, alpha-blockers, beta-blockers, DHP CCBs, non-DHP CCBs, vasodilators, ACE inhibitors, and ARBs. Patients using more than one type of antihypertensive drug in the 180 days before the date NOD was diagnosed were categorized as combined users. Patients who had used antihypertensive drug within the previous 6 years, but not within 180 days before the date NOD was diagnosed were excluded from the analyses. Finally, we excluded 17 patients who were lost to follow-up or died. A total of 1,001 patients with hypertension only were selected for this study. This study was approved by ethics committee of China Medical University Hospital.

### 2.3. Drug Classes

The antihypertensive drugs were categorized into 8 drug classes (thiazide diuretics, alpha-blockers, beta-blockers, DHP CCBs, non-DHP CCBs, vasodilators, ACE inhibitors, and ARBs). There are 6 drugs in the alpha-blocker class, 5 drugs in the ACE inhibitor class, 5 drugs in the ARB class, 10 drugs in the BB class, 6 drugs in the DHP CCB class, 7 drugs in the non-DHP CCB class, 5 drugs in the thiazide diuretic class, and 10 drugs in the vasodilator class in the China Medical University Hospital.

### 2.4. Statistical Analysis

Continuous variables are presented as mean ± SD. They were compared by the Welch *t*-test. Categorical and discrete variables are presented as frequencies and percentages. When appropriate, they were compared by either the Fisher's exact test or the chi-square test. This study aimed to find out what drug classes might increase or decrease the incidence probability of developing NOD. The 8 drug classes are the main effects adjusted by total drug days (tdays). Logistic regression analysis was applied. The odds ratio was used to measure the incidence probability of NOD. The Wald confidence interval for odds ratio (*θ*) was used to define the significant difference under *α* = 0.05. If the confidence interval for *θ* contains 1.0, it is plausible that the true odds of developing NOD are equal among drug classes. If it is greater than 1, the probability of developing NOD among patients who took this drug class is higher than that among patients who did not take that class of drug. An odds ratio less than 1 indicates that the drug class has a low probability of being associated with the development of NOD. Finally, multiple logistic regression models including sex, age, current smoking, familial history of DM, BMI, total cholesterol, triglyceride, LDL cholesterol, HDL cholesterol, and fasting blood sugar were implemented. All analyses were performed using Statistical Analysis software, version 9.1 (SAS). A two-tail *P*  value < 0.05 was interpreted as significant.

## 3. Results

### 3.1. Baseline Data of All Patients

Of the 1,001 eligible patients from January 2006 to December 2011, 120 (12.0%) patients developed NOD.  Table 1 describes the baseline characteristics of study participants of the two groups. Patients ranged in age from 31 to 81 years; the mean age for NOD patients was 59.8 years and that of non-NOD patients was 56.4 years. There were significant differences in age between these two groups of patients (*P* = 0.003). Men comprised less than half (447, 45%) of the sample population.

There were no significant differences in current smoking, blood pressure, and familial history of DM between these two groups of patients (*P* > 0.05). BMI, total cholesterol, Triglyceride, LDL cholesterol, and fasting blood glucose of the NOD group were significantly higher than the non-NOD group (*P* < 0.05). Only there was no significant difference in serum creatinine and HDL cholesterol of lipid profile between these two groups of patients (*P* > 0.05).

Approximately 23% (234) of the patients took only one drug class, 35% (350) took two drug classes, 34% (342) took three drug classes, and 7% (2,353) of patients took from four to five drug classes (Table 1). Over half of the patients took DHP CCBs (59%) beta-blockers (54%). Only 8% (76) of the patients took non-DHP CCBs. The distributions of prescription thiazide diuretics, alpha-blockers, ACE inhibitors, ARBs, and vasodilators are shown in Table 1.

### 3.2. Multiple Logistic Regression Results after Adjusting Sex, Age, Current Smoking, Familial History of DM, BMI, Total Cholesterol, Triglyceride, LDL Cholesterol, HDL Cholesterol, and Fasting Blood Glucose

The risk estimate of NOD after adjusting sex, sex, current smoking, familial history of DM, BMI, total cholesterol, Triglyceride, LDL cholesterol, HDL cholesterol, and fasting blood glucose for users of thiazide diuretics (OR, 1.65; 95% confidence interval [CI], 1.12–2.45) and non-DHP CCBs (OR, 1.96; 95% CI, 1.01–3.75) was significantly higher (*P* < 0.05) than for nonusers. Beta-blockers, DHP CCBs, alpha-blockers, ACE inhibitors, ARBs, and vasodilators were not associated with increased risk of NOD (*P* > 0.05) (Table 2).

## 4. Discussion

The present study demonstrates that thiazide diuretics and non-DHP CCBs were independently associated with an increased risk of NOD in a clinical setting. The use of alpha-blockers, beta-blockers, DHP CCBs, ACE inhibitors, ARBs, or vasodilators was not associated with NOD. 

The present results differ from those of our previous report on beta-blockers, ACE inhibitors, ARBs, and alpha-blockers [11]. However, these differences may be due to the relatively younger age (53 versus 68 years) of participants, different study periods, relatively smaller sample size (1001 versus 24688), and different population of patients taking these classes of antihypertensive drugs in this study. Nonetheless, both studies demonstrate that vasodilators are not associated with a risk of NOD.

Two differences between the results of our previous study and those in this study need to be emphasized. Firstly, in this study, we only collected thiazide diuretics that have been reported to accelerate NOD in patients with hypertension [14, 15]. Our results are comparable with those reported by Taylor et al. who studied the risk of NOD in three cohorts of 74,186 patients taking different classes of antihypertensive drugs [16]. They found that the relative risk of NOD in individuals taking thiazide diuretics was 1.20 (95% CI, 1.08–1.33) in the cohort of older women, 1.45 (95% CI, 1.17–1.79) in younger women, and 1.36 (95% CI, 1.17–1.58) in men. Many meta-analyses have also confirmed that diuretics increase the incidence of NOD compared with other antihypertensive drug classes [17, 18]. This could be a dose-related effect as higher doses are more likely to be associated with NOD [19]. A change in serum potassium levels could be a possible mechanism for thiazide-induced NOD. In one study, a large potassium infusion increased insulin release by two to three times above the basal levels [20]. This is an important mechanism of potassium disposal because insulin can induce increased cellular uptake of potassium. Secondly, we further determined the effect of DHP and non-DHP calcium channel blockers on NOD in a clinical setting. Calcium channel blockers are generally considered to have mild or no impact on the risk of NOD [16]. Many meta-analyses have indicated that CCBs are associated with a greater increase in NOD than ACE inhibitors and ARBs but a lower increase in NOD than beta-blockers and thiazide diuretics. However, no data has effectively demonstrated the effect of DHP and non-DHP CCBs on the development of NOD. To the best of our knowledge, our study is the first to show that non-DHP CCBs are associated with an increased risk of NOD, while DHP CCBs are not. Two recent meta-analyses showed that CCB therapy is not associated with an increased risk of NOD compared with placebo [15, 18]. The mechanism underlying the increase in NOD in patients with non-DHP CCB therapy, observed in the current study, has not been identified [18]. There is only the report suggesting that the DHP CCB azelnidipine may influence inflammation and oxidative stress indirectly and have a beneficial effect on glucose intolerance and insulin sensitivity in nondiabetic patients with essential hypertension [21]. From our study, it showed significant difference of BMI, total cholesterol, Triglyceride, LDL cholesterol, and fasting blood glucose between these two groups of patients. Probably, non-DHP CCBs therapy have related to metabolic disturbance.

Padwal et al. evaluated 76,176 patients with hypertension and reported that the use of beta-blockers was not associated with NOD [22]. However, experts have commented that the study had a mean follow-up time period of less than one year. Furthermore, theirs was an observational community study, and therefore, the results may have lacked the statistical power necessary to demonstrate an association between that class of antihypertensive drugs and NOD [23]. Our study excludes those factors, and the results further demonstrated the same findings as that report.

Recent studies have indicated that ACE inhibitors and ARBs reduce the risk of developing NOD when compared with the results for other classes of antihypertensive agents [24, 25]. In the current study, both ACE inhibitors and ARBs were found to be unassociated with NOD in patients with hypertension. Our result is the same as that from the Diabetes Reduction Assessment with Ramipril and Rosglitazone Medication (DREAM) trial [26], which failed to show a statistically significant reduction in NOD with the ACE inhibitor ramipril versus placebo in patients with IFG. Another study, Telmisartan Randomized Assessment Study in ACE Intolerant Subjects with Cardiovascular Disease (TRANSCEND) [27] had findings similar to our result that the ARB telmisartan cannot reduce the incidence of diabetes.

In the current study, alpha-blockers and vasodilators were found to be unassociated with NOD. Numerous studies have consistently demonstrated that alpha-blocker classes of antihypertensive medications have differential effects on carbohydrate and lipid metabolism in humans [28]. Our result is different from that of our previous report on the alpha-blocker classes [11]. However, the difference could have been due to the relatively younger age (53 versus 68 years), different study period, and different population of the patients taking this class of antihypertensives in our more recent study.

Some limitations in this study need to be emphasized. First, this was a retrospective and descriptive study in CMUH over a period of six years. Also, we performed analyses that excluded participants with untreated, ongoing hypertension, so caution must be exercised in interpreting our data. Second, all cases in this study were collected from medical records, and diagnoses were based on physician reporting only in CMUH; therefore, it is not clear how our findings can be generalized to patients in different areas. Third, this is a descriptive study and no data regarding time of administering the antihypertensive drugs; therefore, it is not the most effective for determining the relationship between antihypertensive drugs and NOD. Furthermore, the process of insulin resistance in this study of patients who developed NOD must have started many years before the diagnosis, and insulin resistance might have coexisted with the hypertensive condition for which the antihypertensive drug was used. In this situation, the temporality and subsequently the causality of the antihypertensive drugs cannot be determined.

## 5. Conclusion

In conclusion, our findings provide some support for the hypothesis that there are differences in the risk of developing NOD among the different classes of antihypertensive drugs. Our results show that patients with hypertension who take thiazide diuretics and non-DHP CCBs are at higher risk of developing NOD than those who take other classes of antihypertensive drugs in a clinical setting. We suggest that doctors do not use non-DHP CCBs for stage 1 hypertension alone.

## Figures and Tables

**Table 1:** Baseline characteristics of all patients.

	NOD	Non-NOD	Total	P* value
	(*n* = 120)	(*n* = 881)	(*n* = 1001)	
Age (year-old)	59.8 ± 11.4	56.4 ± 13.6	56.8 ± 13.4	0.003
Male (%)	50 (42)	397 (45)	447 (45)	0.537
Current smoking (%)	19 (16)	122 (14)	141 (14)	0.496
Familial history of DM (%)	18 (15)	105 (12)	123 (12)	0.338
BMI ≥ 27 (%)	43 (36)	202 (23)	245 (24)	0.002
Systolic blood pressure (mm Hg)	148.7 ± 10.2	149.0 ± 9.8	149.0 ± 9.9	0.824
Diastolic blood pressure (mm Hg)	77.8 ± 6.2	77.8 ± 6.0	77.8 ± 6.0	0.892
TC (mg/dL)	227 ± 28	219 ± 35	220 ± 34	0.005
TG (mg/dL)	152 ± 52	130 ± 45	133 ± 51	<0.001
LDL-C (mg/dL)	138 ± 30	131 ± 36	132 ± 35	0.019
HDL-C (mg/dL)	58 ± 13	60 ± 17	60 ± 16	0.114
FBG (mg/dL)	101 ± 10	85 ± 12	87 ± 12	<0.001
Serum creatinine (mg/dL)	1.16 ± 0.32	1.13 ± 0.33	1.13 ± 0.33	0.351
Number of prescription (%)				0.040
1	29 (24)	205 (23)	234 (23)	
2	42 (35)	308 (35)	350 (35)	
3	40 (33)	302 (34)	342 (34)	
4	8 (7)	56 (6)	64 (6)	
5	1 (1)	10 (1)	11 (1)	
Drug class				
Thiazide diuretics (%)	38 (32)	215 (24)	253 (25)	0.054
Beta-blockers (%)	76 (63)	518 (59)	594 (59)	0.399
DHP CCBs (%)	68 (57)	474 (54)	542 (54)	0.528
Non-DHP CCBs (%)	13 (11)	63 (7)	76 (8)	0.102
Alpha-blockers (%)	15 (13)	148 (17)	163 (16)	0.233
ACE inhibitors (%)	17 (14)	132 (15)	149 (14)	0.771
ARBs (%)	37 (31)	289 (33)	326 (33)	0.634
Vasodilators (%)	16 (13)	107 (12)	123 (12)	0.591

*P value between NOD and non-NOD.

DM: diabetes mellitus. BMI: body mass index. TC: total cholesterol. TG: triglyceride. FBG: fasting blood glucose. DHP-CCB: dihydropyridine calcium channel blockers. Non-DHP CCB: Nondihydropyridine calcium channel blockers. ACE: angiotensin converting enzyme. ARB: angiotensin receptor blocker.

**Table 2:** Incidence of odds ratios (ORs) with 95% confidence intervals (CIs) for NOD according to prescriptions for antihypertensive drugs compared with nonuser subjects.

Drugs	Adjusted OR*	Adjusted 95% CI	P** value
Thiazide diuretics	1.65	1.12–2.45	0.012
Beta-blockers	1.39	0.94–2.06	0.099
DHP CCB	1.24	0.84–1.82	0.276
Non-DHP CCB	1.96	1.01–3.75	0.044
Alpha-blockers	0.71	0.31–1.68	0.445
ACE inhibitors	1.53	0.90–2.64	0.117
ARBs	1.16	0.77–1.75	0.470
Vasodilators	0.92	0.53–1.60	0.777

*ORs were adjusted for age, sex, current smoking, familial history of DM, BMI, total cholesterol, Triglyceride, LDL cholesterol, HDL cholesterol, and fasting blood sugar.

**P value between users and nonusers.

DHP-CCB: dihydropyridine calcium channel blockers.

Non-DHP CCB: nondihydropyridine calcium channel blockers.

ACE: angiotensin converting enzyme.

ARB: angiotensin receptor blocker.
